# Exploring the impact of a community participatory intervention on women's capability: a qualitative study in Gulu Northern Uganda

**DOI:** 10.1186/s12905-020-01170-8

**Published:** 2021-01-18

**Authors:** Loubna Belaid, Emmanuel Ochola, Pontius Bayo, George William Alii, Martin Ogwang, Donato Greco, Christina Zarowsky

**Affiliations:** 1 Family Medicine Department, McGill University, 5858 Chemin de la Côte des Neiges, Montreal, QC Canada; 2 St. Mary’s Lacor Hospital, P.O. Box 180, Gulu, Uganda; 3 International Prevention Research Institute, Allée Claude Debussy, 69130 Écully, Lyon, France; 4 University of Montreal, 7101 Av du Parc, Montréal, QC H3N 1X9 Canada

**Keywords:** Women's capability, Qualitative study, Reproductive, Maternal and child health, Community mobilization, Gender norms, Participatory learning action cycle, Northern Uganda, Post-conflict setting

## Abstract

**Background:**

Community participatory interventions mobilizing women of childbearing age are an effective strategy to promote maternal and child health. In 2017, we implemented this strategy in Gulu Northern Uganda. This study explored the perceived impact of this approach on women's capability.

**Methods:**

We conducted a qualitative study based on three data collection methods: 14 in-depth individual interviews with participating women of childbearing age, five focus group discussions with female facilitators, and document analysis. We used the Sen capability approach as a conceptual framework and undertook a thematic analysis.

**Results:**

Women adopted safe and healthy behaviors for themselves and their children. They were also able to respond to some of their family's financial needs. They reported a reduction in domestic violence and in mistreatment towards their children. The facilitators perceived improved communication skills, networking, self-confidence, and an increase in their social status. Nevertheless, the women still faced unfreedoms that deprived them of living the life they wanted to lead. These unfreedoms are related to their lack of access to economic opportunities and socio-cultural norms underlying gender inequalities.

**Conclusion:**

To expand women's freedoms, we need more collective political actions to tackle gender inequalities and need to question the values underlying women's social status.

## Background

Community-based women's groups using a participatory learning and action (PLA) cycle are an effective strategy to promote maternal and child health [1, 2]. A trained female facilitator discusses mothers' and children's health issues with women from her community. This approach is anchored in Freire's philosophy, which proposes that marginalized communities can mobilize themselves and take collective actions to address poverty and social issues [3]. There is robust evidence that the approach can reduce maternal and newborn mortality rates, can improve health-seeking behaviors, and has psychological and social benefits [4–6]. Studies have described an increase in women's knowledge, capacity, and self-confidence to adopt safe and healthy behaviors [7–9]. Women's groups in Nepal enabled social support and social networks that strengthened community agency [10]. A 2019 mixed-methods systematic review of community mobilization interventions reported participation, shared information, social cohesion, collective action, critical consciousness, knowledge, and self-concept as potential mechanisms underlying such interventions' impact. The review authors identified enablers and barriers at the community (poverty, power hierarchies, health beliefs) and intervention levels (staff management, incentives, educational tools, inclusion of most marginalized sub-populations), but they did not have high confidence in any of the mechanisms, enablers, and facilitators identified. They highlighted the limited evidence of impact on women's capability and gender dynamics [11]. The review identified only three studies assessing the PLA's effect on women's agency and gender dynamics [12–14]. Two studies from Nepal, using the relative autonomy index (RAI) to measure agency, did not find impact of the PLA intervention on women's agency in the household [12, 13]; there was limited evidence of increased agency through group participation [12]. Participatory women's groups in rural Bangladesh did not report an increased in women's household decision-making about their healthcare [14].

The three studies assessing impact of PLA on agency and gender dynamics used quantitative study designs and were conducted in South Asian countries. Qualitative research has the potential to explore contextual factors interacting within the change behavioral pathways. We are not aware of published studies assessing the impact of the PLA approach on women's capability and gender dynamics in Africa. While women's empowerment and gender equality are Sustainable Development Goals, we need more evidence to inform public health interventions and policies to reach these ultimate goals. This study aimed to explore the perceived impact on women's capability of women's groups practicing the PLA cycle to promote maternal and child health in Gulu, Northern Uganda.

### Study setting and intervention

The study took place in Gulu district (2014 population estimate 425,094), located in Northern Uganda [15]. The population is mainly rural and Catholic and depends on subsistence-based agriculture. The main ethnic group is Acholi (Luo speaking Nilotic population) with a historically highly democratic, patrilineal clan/ lineage-based political organization [16]. Northern Uganda region went through two decades of civil war (1986–2006) between the Lord's Resistance Army and the Uganda military. Over 1.8 million people were internally displaced [17]. As a result, the region failed to make progress in maternal and child health. Gulu district has one of the highest burdens of unmet need for family planning, neonatal mortality, teenage pregnancy, and child mortality in Uganda [18].

In 2016, we assessed the feasibility of implementing a women's group intervention using the PLA cycle to promote maternal and child health. The intervention encouraged health promotion activities and community mobilization. In 2017, we implemented the intervention. To avoid creating parallel community structures, we integrated the PLA approach into 12 pre-existing savings groups. Savings groups are small groups where members pool their savings contributions and then borrow from them to improve their members' access to financial support. Women engage in these groups to pay for medical expenses, pay school fees for their children, expand their farming activities, or start a new income generation activity.

The 12 groups were distributed in three areas within the Gulu district: Opit (rural), Amuru (remote), and Gulu (semi-urban). We invited the groups to select members to facilitate the meetings using the PLA cycle. We trained two women from each group as facilitators for five days (total 24 women), with a three-day refresher training in 2018. A local expert on the participatory PLA approach facilitated both trainings. We hired three female supervisors (one for each area, in charge of four groups each) to support the facilitators. The supervisors attended each group meeting.

Usually, the savings groups met monthly, but the groups took the initiative to meet weekly during our intervention. The PLA meetings followed a four-phase cycle: (1) identify and prioritize problems that may occur during and after delivery and during childhood (2) plan activities to reduce these problems; (3) implement strategies to address the priority problems; (4) assess the activities [23]. In phase 3, the facilitators invited men and other community members to discuss the implementation of the strategies to address the problems identified in phase 1. They used picture cards to facilitate the meetings. The cards represented the most common maternal and child health problems and guided strategies to manage these problems. The project provided each group with 30US$ monthly to add to their savings. The intervention ran from January 2017 to March 2020, and data collection took place in October 2019.

### Conceptual framework

There is a growing interest in using Sen's capability approach to assess health policies and programs [19–21]. Scholars have emphasized the relevance of using this approach to evaluate reproductive health programs in low-and-middle-income countries [22, 23].

The capability approach is a theoretical framework for assessing well-being, development, and justice. The framework represents a paradigm shift from assessing well-being through utility and preferences to examine *people's capabilities*, meaning the *real freedoms* people have to live the life they want to live [24]. The concept of capabilities is the ability to achieve the "doings" and "beings" that people have reason to value in life [21]. Those "beings" and "doings" can range from *basic functioning* such as being well-nourished and having decent housing to more *complex functioning*, such as having control over personal decisions [21]. Conversion factors influence these functionings. Sen identified three types of conversion factors: individual (personal characteristics: age, mental, physical health), social (social, cultural norms of a society), and environmental (physical environment) [24].

**Fig. 1:**
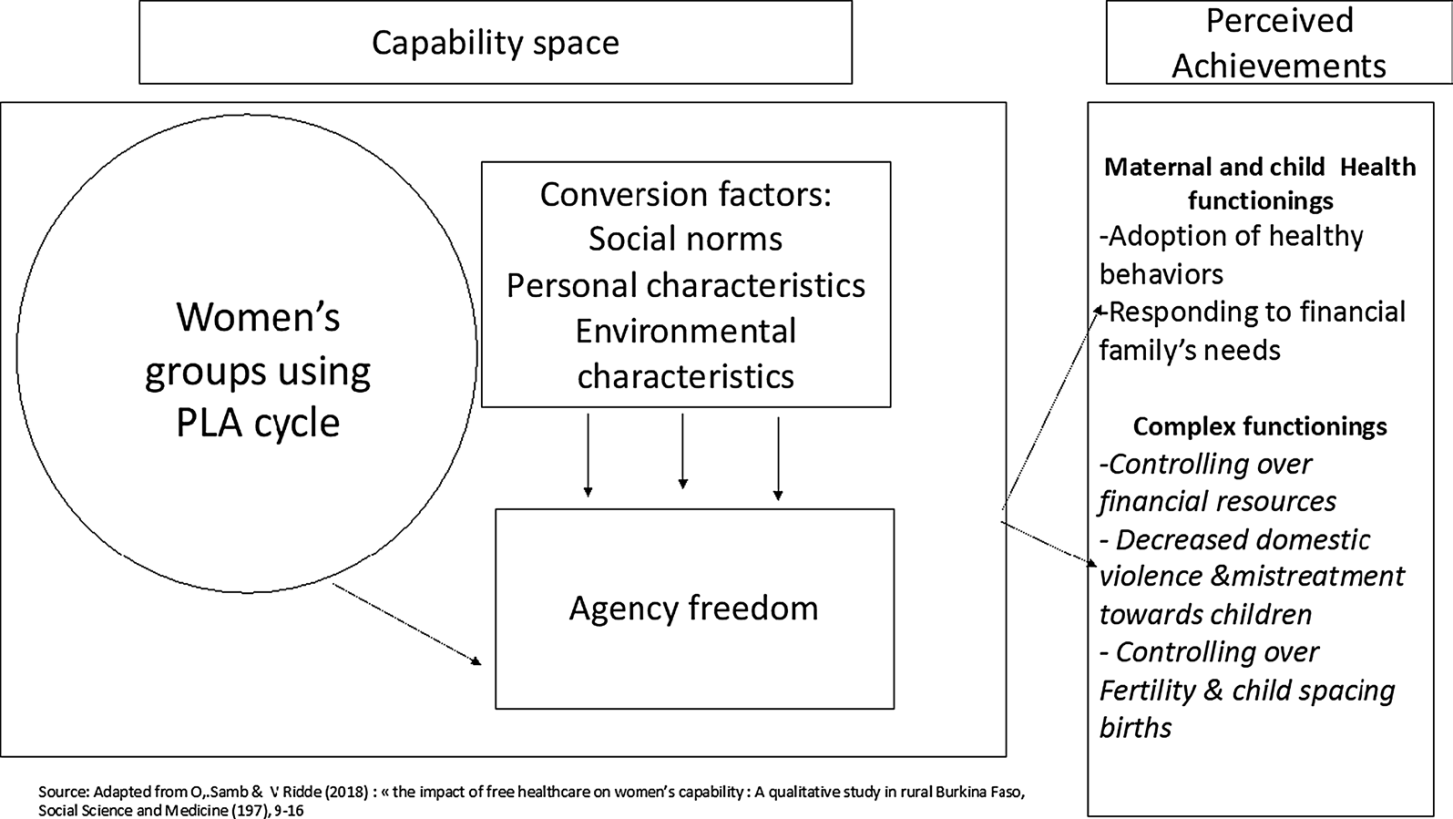
Framework

In this paper, we explore how the participatory women's groups intervention enabled women to achieve capabilities in the area of maternal and child's health from *basic health functioning* to more *complex functioning* (controlling financial resources, decisions on fertility and child spacing practices, individual and collective actions to reduce family violence). We consider the women's groups using the PLA cycle as a resource introduced into women's capability space, and we consider the influence of conversion factors on women's agency freedom (Fig. 1). We used this framework because it acknowledges that people differ in how they shift a resource into a valuable achievement and because it captures gender inequalities.

## Methods

### Study design and data collection methods

We conducted a qualitative study based on in-depth individual interviews, focus group discussions (FGDs), and document analysis.

### In-depth interviews and focus group discussions

We followed COREQ guidelines for reporting interviews and focus groups discussion [25].

A team member, a female anthropologist, designed the interview and FGD guides for this study. The interview and FGD guides developed for this study are provided as Additional files 1 and 2. The interview guide reflected the research question and the theoretical framework. It covered the following items: perception of the PLA intervention, its impact, resources, and agency to seek health for themselves and their children, reproductive health (number of children desired, child spacing practices), access to resources, division of labor, social and cultural values. The FGD guide included the perception of the PLA intervention and its impact at individual and community levels.

A local male coordinator trained in qualitative research and experienced working with rural communities translated the interview and the FGD guides from English to Acholi. The male coordinator selected eight groups from among the 12 PLA groups, reflecting the spread of rural, remote, and semi-urban locations across Gulu district. We further selected for interview those participants who regularly attended the group meetings among the eight groups. The facilitators from all the 12 PLA groups participated in the FGDs.

Two trained team members (female and male) familiar with customs and traditions conducted the individual interviews and the FGDs. The interviews aimed to explore the impact of the intervention on individual group members' lives, and the FGDs described the effect of facilitating the groups on the facilitators' role in the community and the impact at the community level. The FGDs did not cover personal questions. The facilitators were comfortable sharing their individual experiences since they were familiar with each other. All the participants agreed to be part of this study.

The interviews and FGD lasted about an hour and were in the Acholi language. The fieldworkers conducted the interviews at group members' homes and the FGDs in a public place. We digitally recorded interviews and FGDs. We continued collecting data until we reached data saturation.

### Document analysis

We examined reports from the coordinator and the supervisors of the women's groups. The supervisors wrote reports of each group meeting. These reports included the groups' daily activities, the challenges, the opportunities to manage their groups (mobilization, participation, engagement), and their achievements (actions to tackle maternal and child health problems, resources mobilization, organization of their actions).

### Data management and analysis

We transcribed and translated the recorded interviews and FGDs from Acholi to English. The female anthropologist used a hybrid (deductive and inductive) approach to build the coding structure and coded the interviews and the FGDs transcriptions with Nvivo 11 software.

We triangulated the data between the sources and data collection methods to identify similarities and differences and looked at convergence patterns to corroborate interpretation [26, 27].

We analyzed the documents manually. The coordinator synthesized the problems prioritized and the actions taken by the groups in a table.

The co-authors and the project team members (coordinator, supervisors) discussed the data interpretation, including reflexivity [26]. We reflected on how our characteristics (gender, ethnicity, religious background, social status (researcher, being part of the project team)) might have influenced the data collection and analysis. We discuss the potential biases and internal and external validity of our study in the limitations section of this paper.

### Ethics approval

This study respected the principles in the Declaration of Helsinki. We followed additional guidance from the ethical principles for research in post-conflict settings [28]. We obtained ethical clearance from Uganda and Canada. The participants gave their consent to participate. We treated all information from participants as confidential.

## Results

### Maternal and child health functioning achieved

#### Adoption of safe and healthy behaviors

The women's group intervention enabled women to adopt safe and healthy behaviors to protect their health and their children's health at individual and community levels. By discussing and reflecting on knowledge about the root causes of health problems, women realized their ability to prevent common diseases affecting maternal and child health. As illustrated in the following quotes, women frequently mentioned how the knowledge they gained from the intervention was key to triggering their behavior change. The quotes reflect the most common topics discussed in the meetings including malaria, sanitation, heavy work during pregnancy, and health services use during pregnancy and childbirth.

I learned so much and realized that we could fight some of these diseases that disturb us so much, for example, malaria (IDI#8). "I gained knowledge and skills in managing and preventing malaria. I was able to start attending antenatal care and other healthcare services earlier (participant #1, FGD O).

"An example is malaria, which we learned that we could curb by slashing the bushes around our homes. I also learned that the toilets should be kept clean and we should sleep under mosquito nets" (ID#8). "I learned that when I have a problem like bleeding, or anything is happening to me when I am pregnant; I am supposed to rush to the hospital and not anywhere else” (ID#8).

The group teaches us how to take good care of ourselves during pregnancy. For example, we were taught that when you are pregnant, we should not do bulky work. We should be in harmony with our husband because disharmony brings so many issues that can be dangerous to your pregnancy (ID#7).

From my observation, this group helps us because we did not know some problems here in our village, which we can solve (ID#12).

I stopped doing hard labor because it causes hemorrhage, and if I am to compare the level of hygiene at my home now with the previous years, I find that I am better off now (IDI# 5).

During the focus groups discussions, the facilitators strongly emphasized the realization of their ability to improve their health. They used the concept *Medo Kero,* an Acholi concept that means the ability or power to do something on our own. The women's group intervention helped the facilitators to understand that most diseases are preventable. They reported their previous lack of awareness about health promotion activities.

We realized that it is not the hospital who will solve our problems. It is us. We can do something from our own. Now we know, and we are empowered (participant #2, FGD O).

Before this project, people had blind eyes. We did not have enough knowledge. In this world, if you want to be supported, you will get it by yourself because people will get tired to support you. We realized that what we do by ourselves is more sustainable (participant # 3, FGD O).

Our government was not using this approach [prevention], the problem can be prevented by the source (participant #4, FGD 0).

At the community level, the women's group intervention enabled the women to implement collective actions by providing social and financial support and mobilizing internal resources. Most of the groups identified malaria as a problem for mothers and children. To tackle this problem, each group implemented several strategies such as community sensitization, clearing bushes, and home visits to check if household members were sleeping under mosquito bed-nets. Other groups tackled access to health care services. One group mobilized funds to buy a motorcycle and lobbied for extra funds from their local political leader. Another group mobilized resources and built a bridge over a river that impeded access to public services (health facility, market, schools) (Table 1).

We have been supporting each other to seek medical care when our children are sick. There has been improved sanitation and hygiene because some percentage of liquid soap made is given to group members (ID#4).

These days, all the children sleep under treated mosquito nets and therefore, there are no common cases of malaria at my home like it was before (ID#7).

**Table 1:** Problems and actions taken by the groups

Name of the groups	Problems identified	Actions
RM	HIV/AIDs for mothers and malaria in new-borns Poor access to the health centre, school, and market	Work on a feeder road that connects L0 to AM primary school to improve access to school, health centre, and market
TD	Access to the health centres and schools hampered with lack of a bridge to cross a river HIV/AIDs in mothers and malaria in children	The group completed a bridge The group shared the MoCHeLaSS intervention with other neighbouring saving groups across the river which made mobilisation for the work easier
KL	Malaria and pneumonia in their first cycle Diarrhoea in the second cycle	They want to transform their open water point into a protected well and they have so far acquired all the materials needed for the construction by themselves
PC	Haemorrhage and malaria in the first cycle Currently working on pneumonia and lack of safe water	Regularly clean water points together Have put in place by-laws against grazing, washing, and bathing at the water point
MK	Malaria for both women and their new-born babies	Community sensitization Completed implementation of strategies; took activities like clearing bushes around homes to destroy breeding grounds for mosquitoes
TA	HIV/AIDS for mothers and malaria for both mothers and children	They carried out community sensitization using plays Convinced the local leaders to make a bye-law against staying out late in the night in order to bring down vices due to the influence of alcohol and other drugs Encouraged members to take HIV tests
OC	Malaria for both mothers and children	Group members moved door-to-door to do sensitization and some enforcement with the help of community leaders on the use of ITNs and hygienic practices such as clearing around homes, filling holes, draining stagnant water
LK	Malnutrition and diarrhoea	Last season the group planted 2 acres of soybeans to help malnourished children and mothers in the village but also act as role model for the other village members to emulate Moved in their villages to check if most households planted the necessary foodstuff and have a clean environment with rubbish pits, dish racks etc
BG	Long distance to the health Centers Malaria	They have continued saving money to buy a motorcycle Lobbied the sub-county leaders and got some funds to add on their savings for the motorcycle Community sensitization campaigns on prevention and management of malaria
WA	Haemorrhage brought by malaria	They carried out community sensitization in their community on malaria with the help of Lacor health educators Have been lending money to vulnerable individuals in their communities to access health care Have shared MoCHeLaSS initiatives with other 2 savings groups in their community
RT	Haemorrhage Malaria	Community sensitization and referrals They formed support groups to do home visits and counselling They engaged the hospital to improve on its lagoon and incinerator which they believed was affecting their community (overflowing, mosquitoes, bad odour)
NW	Adolescent pregnancy Haemorrhage and diarrhoea	Community sensitization and giving skills such as making liquid soap and eco-charcoal (from used card boxes) to adolescent girls Encouraging adolescents and child-mothers to join the group Were able to bring on board 12 adolescents/child-mothers trained them on how to become self-reliant and take care of themselves and their babies

#### Responding to personal and family financial needs

Through their savings and the intervention's financial contribution, women could respond to some of their financial needs.

We have been engaged in income-generating activities in our group. For instance, we make liquid soap and charcoal, which we sell and share the profits. This has enabled us to move on in life and stand on our own. We can earn some money. When our children fall sick, we can take them to the health facilities without seeking support from other people. We do not have to go out to the streets to beg (IDI#4).

One group offered additional financial capital to teenage mothers wishing to start an income-generating activity. In the following quotes, three teenage mothers described what they could achieve with this new financial resource. Financial achievement included responding to basic needs, such as having good food, having a decent house, and accessing education and health services. Women considered having the ability to take care of themselves and their children as an outstanding achievement in their lives.

"I can eat every-day; I used to have one meal per day. When my child is sick, I can bring her to the hospital" (IDI#9). "It is still in process, but I am building my own house. I am paying for my sister's school fees" (IDI#7). "I started to do some business with the money. Every day, I sell dry fish and tomatoes. I can take care of my child and myself. I got advice about how to do business. I am saving money to pay for my school fee. I plan to go to a tailoring school" (IDI# 3).

"The money that I save in this group is what makes me proud. I want to pay my children's school fees and become doctors in the future. I also want to relieve my husband of some of the burdens like paying school fees so that he does not have to do everything by himself" (IDI#2)." I want my children to study and get good jobs in the future and be independent and not become thieves (IDI#7). "I feel strong, I can buy my clothes, and I can also make my own decisions. I want my child to study as far as possible” (IDI#3).

### Complex functioning achieved

Women achieved other, more complex functioning. They extended their agency to control their financial resources and developed strategies to stop domestic violence. As a result, women saw a reduction in mistreatment of children. For some women, they extended their agency to control their fertility and child spacing practices.

#### Control over financial resources

Women used three strategies to control their financial resources: bargaining, hiding, and resistance. The following quotes illustrate each strategy:

A woman negotiated with her husband to pay for the school fees of her child that she had before marrying him: "I was telling him. C [name of the husband] you find me with this child, you said you would help me with this child. Please help me with his school fees; my money is too little to handle it" (IDI#13). "If he asks for my money, I will say to him that it is not there" (IDI#9).

I hide from my husband about my savings. Some men ask for money from their wives saying they would return it with some profits, but this is sometimes not the case (IDI#2).

We spent equally. If he asks for my money. I will ask him what he is going to do with it. If he is going to do bad things. I will say no to him (IDI#7).

#### Decreased domestic violence

As a result of the post-conflict situation, Gulu region has a high prevalence of intimate partner violence [29, 30]. Women discussed domestic violence, its root causes, and its consequences during their group meetings. The intervention enabled creating a safe space where women who suffer from domestic violence shared their experience to seek support from the group. Women exchanged advice on how to communicate and discuss with their spouses. Within the PLA cycle, the third phase included men. In this phase, the group explained the problems and the strategies to address them to the men and invited them to join the group members to implement the strategies. Some women also asked if the facilitator could intervene to counsel them with their spouse as a couple. The invitation of men at phase 3, the facilitators acting as a counsellor, and the advice sharing among the group members contributed to reducing domestic violence. Women saw this outcome as a significant change that they attributed to the intervention.

"I noticed less domestic violence and less quarreling, and now there is more respect between me and my husband" (participant #1, FGD A). "Mochelass [name of the project] has improved the attitude of my husband, and now he has reduced alcohol consumption" (participant #2, FGD A).

I did not know how to manage my domestic violence. Now, I know how to handle this issue and advise others about it (participant #1, FGD G).

He used to be rude to me, the way he talked to me. He did not give me the money when the baby was sick. After the group invited the men, he came back with some ideas. I started my own business and advised him to start a job. He sells utensils in the market, and he also has left the other girls (IDI#7).

Mochelass teaches us about married life, and I wanted to learn about these because I know no one will come from somewhere else to teach me about married life. The older women in the group also give us good pieces of advice. I also learned that a woman whose husband drinks a lot of alcohol should also know how to calm him down when he gets drunk. Most of the women in this area whose husbands are drunkards are very tough and rude. They always quarrel a lot with their husbands. Last week, the group gathered together and advised on how to handle these husbands (IDI# 1).

We reduced domestic violence; when someone has this problem, we share with the group. We have seen that this problem has slowed down (participant # 2, FGD G).

#### Reduced mistreatment of children

The perception of a reduction in violence towards children was an unexpected positive impact of the intervention. Discussing and reflecting on domestic violence had a positive ripple effect on mistreatment of children.

"I learned a lot. I should not be rude. I used to beat my children. With mochelass, I know how to talk to them" (participant # 1, FGD G). "My attitude has improved. I have softened my character with my children" (participant # 2, FGD G). "We used to put all our anger from our husbands on our children" (participant #4, FGD G).

#### Number of children desired and child spacing practices

We did not find a clear-cut answer about whether the intervention enabled women to control their fertility and child spacing practices. Few women were using contraceptive methods. Some women reported that they decided on their own about their fertility. Others indicated that it was a joint decision, and their spouses supported their decision to use contraceptive methods. One woman did not want to have more children but could not directly confront her husband. She pretended to be sick and took "fake" medications for months. The husband put pressure on her; she finally gave in and became pregnant with her fourth child. Socio-economic situations and the quality of their relationships with their husbands influenced women's control over their fertility and child spacing practices.

When we had just started staying together, we discussed about the number of children. He proposed that we have four children, but given this challenging time, I proposed that we have three children, and that is how our discussion still stands up to date (ID#7).

I want to have six children, and my husband wants four. I have been using implants for three years. He did not say anything about it [using implants] (ID#8).

Looking at the fact that education has become very costly, I decided that I should bear a maximum of four children. I do not want to bear too many children whom I would only punish with no education. I made this decision alone. My husband does not give me any support even when I am pregnant and also does not pay the children's school fees, so it is unrealistic to bear too many children with him (ID#8).

#### Other complex functioning achieved by facilitators

For most facilitators, their participation in this intervention increased their communication skills, networking, self-confidence, and social recognition within their communities. They associated these social and psychological concepts with their well-being and happiness.

"I feel I am a medical person because I can help my friends in need. I feel very happy" (participant #2, FGD G). "I got a new title from the villagers as the healthcare provider" (participant #4, FGD O).

"I improved my ability to talk to people. Before I was not able to talk. Now, I can listen and mix with different people" (participant # 1, FGD G). "I used to be fearful, and I feel strong now" (participant #3, FGD G).

"Before this project, I could not speak as I can now. Even if the president comes here, I can ask him a question without fear" (participant #1, FGD O). "I can invite all the leaders because I know all of them" (participant #2, FGD O). "I gain confidence in the community" (participant #3, O). "I gained confidence in talking in public" (participant 1#, FGD G). "I gained confidence in speaking in front of people" (participant #2, FGD A). "I can enter any house, and I can interact with big doctors" (participant #5, FGD G).

### Social and cultural conversion factors impede women's freedoms

Although the intervention contributed to increasing women's capabilities in some areas of their lives, some social and cultural norms limited their full agency freedom. Accessing and owning assets, gendered division of labor, and women's status are obstructions to increasing women's capabilities.

#### Accessing and owning assets

In Acholi culture, men control access to land, livestock, and crops [31]. The land is a major asset for subsistence and a source of income. Women access to land only through their husband's kin. A woman loses access to the land if she divorces or becomes a widow and has no sons [32, 33]. The two decades of conflict led the Acholi people to lose their lands and livestock. This created important land conflicts in modern Acholi communities, increasing pressure on limited resources and putting women in a more vulnerable position. Although women contribute to working the land and do some activities to generate income, men remain the main providers. This status gives men more power over the household's decision-making process. The following quotes described this power over household decisions. Women perceived their financial contribution as limited and this led them to be more dependent on their spouse's financial resources.

It would have been good if I could do some work so that I could earn money as my husband does. I might want my child to study in some school, and my husband might want him to study in another school, and this brings tension, but in most cases, the decision comes from him because it is him who pays the school fees (IDI#12).

"He is not supporting me, being in the group. He gives me time to participate, but the decision is quite hard. For most things, he is the one who decides. He accepts few of my ideas. I still need to consult him" (IDI, #13). "I cannot be above him. He is the one who decides. I can only advise" (IDI#7).

Men are highly valued in the Acholi culture. When one has many female children and only one male child, all the riches and wealth of the home is assumed to be for the male child (IDI#1).

We had a garden, and my husband didn't want us to work on it that year. He wanted us to leave it till next year. Still, I wanted us to dig it and plant some maize in it that year and plant whatever he wanted the following year. Still, he refused, He hired out the land to other people, and that annoyed me so much, but I decided to leave him alone to think about the issue and see whether he did the right thing or the wrong thing, and I chose not to talk to him about that issue again (IDI#4).

#### Unequal gendered division of labor

Before the armed conflict, women were engaged in farming activities such as planting groundnuts, sesame, maize, and beans, and men were in charge of cultivating cash crops (rice, cotton, cassava, sorghum). Men were in charge of heavy work, such as clearing and preparing agricultural land. The conflict disrupted labor division by preventing men from fulfilling their roles, and women assuming their roles, and also men's role as providers [33]. Women perceived the division of labor as unfair, but they accepted it against their will.

It is not fair. Women are cheated because they do most of the work both outside and within the household (ID#4).

In the following quotes, women described the lack of spouse involvement in household's tasks:

Normally, husbands do not accept, but I would wish my husband to help me fetch water and go to the market to buy food and wash clothes on days that I am weak (ID#7).

I feel that I do more work than my husband because there are times when he goes for work, and there are no customers, so he comes back and just sits at home while waiting for food (IDI#7).

Here in the Acholi culture, the men do not do work like cleaning and cooking. I might ask him, but I know he would not help me. Most Acholi men have many women, so they would just go to another woman when you ask them to work for you (IDI#8).

#### Women's status

Asked about their value in their communities, women highlighted their fertility, their capacity to work, to care and love, and to respect their spouses and family members. These values are intrinsically linked to ensuring the well-being of others.

"A woman should be reproductive by being able to bear children, productive by working hard in the gardens as well as at home and should be loving to the people" (IDI#4). "A barren woman is not accepted in a home (IDI#1). A good woman should be fertile, hardworking, especially in the garden, hospitable, loving, and kind to people" (IDI#1). "The woman should be hardworking, should love the people and take good care of them, for example, the elderly. She should generally be hardworking whether in the garden or doing office work" (IDI#8). "A good woman must be respectful, obedient, and listens to the husband" (IDI#5).

## Discussion

The Sen capability framework allowed us to explore what women *achieved* (adoption of safe and healthy behaviors, freed from domestic violence), *partly* *achieved* (control financial resources, fertility, spacing births) and what *they strived for* (accessing economic opportunities, owning assets, and equal gender division of labor). The facilitators perceived improved communication skills, networking, self-confidence, and an increase in their social status. The framework shed light on social and cultural conversion factors (women's status and cultural gender norms) obstructing women's full freedom in the context of implementing a participatory community intervention.

A study using the Sen capability approach to design a quality of life measurement indicator in Malawi identified a list of capabilities that women strive for. The following dimensions are part of the list: avoiding diseases, having enough food to eat, being able to space births, being free from domestic violence, living in a decent house, being able to take care of husband and children, owning assets, able to access business opportunities, being respected, being able to access services [21]. Our findings echoed these dimensions and implied that they could be used in the Ugandan context to design programs to improve and measure women's quality of life.

The adoption of healthy behaviors is consistent with a large body of evidence supporting the positive impact of interventions aiming to increase women's empowerment and agency on maternal and child health outcomes [34–36]. A 2016 systematic review found 67 articles reporting a positive association between women's empowerment indicators and maternal and child health outcomes [37]. A 2019 study based on the analysis of population-based cross-sectional surveys from 59 low-and middle-income countries (n = 6,12,529) showed that women's empowerment is associated with child survival [38]. This positive association between women's empowerment and maternal and child health outcomes raises debates among scholars about the current theory, practice, and measurement of women's empowerment [39–41]. Many scholars recognize the concept's multidimensionality and the challenge of measuring and interpreting its results [37, 40–42]. For Kabeer, this positive association should not be seen as a manifestation of empowerment but rather self-efficacy because children's care is pre-assigned to women's role [40]. For Sen, women's agency is intrinsically linked with the well-being of the households [24]. The study in Malawi and our findings reflect Sen's approach to women's agency [21]. Women strived to improve their health and well-being but also the health and well-being of their families. These findings imply the importance of clarifying the theory and value-based assumption when assessing women's capabilities [42].

Controlling financial resources, decisions around fertility, and child spacing were partly achieved. Our results brought a more nuanced picture than the quantitative studies conducted in Bangladesh and Nepal. The authors did not find evidence on the impact of the PLA approach on women's agency in the household [12–14]. In our study, men remained the primary financial provider, and women were limited in their ability to access economic opportunities. Nevertheless, some women could respond to some of their family's financial needs and tried to extend their agency to control the new financial resources they own. The cultural system underlying access and owning land and livestock in Northern Ugandan will continue to hinder women's functioning through expanding their financial capability. A study in India using the capability approach demonstrated that owning a house or land significantly reduced women's domestic violence risk. Property provides women's economic and physical safety, increases their self-esteem, and opens new opportunities [43]. Another study among displaced tribal women in India reported how the lack of access to agricultural land and forest resources deprived women of fulfilling their capabilities [44].

Domestic violence is a violation of women's dignity and rights, and studies using the capability approach have reported this clearly [21, 23, 43, 44]. The PLA intervention enabled participating to develop individual and community strategies to reduce domestic violence. The strategies that they came up with are clues for designing programs to tackle domestic violence.

Our results around controlling fertility and freedom to practice birth spacing were not clear-cut. While some women seemed to exert agency to decide on the number of children they wanted to have, for others, it was a joint decision, and the husbands supported their decision to use contraceptive methods. Although women knew about the available contraceptive methods, few reported using them. Our finding contrasts with conclusions of a study among displaced tribal women in India, where women did not use contraceptive methods because of their lack of knowledge, awareness, and fear of using health services [44]. Studies in Bangladesh and Malawi did not report changes in family planning outcomes after implementing women's group interventions [14, 45]. A study in Burkina Faso using the capability approach to assess an intervention to remove primary care user fees did not report changes in family planning outcomes [19]. One possible explanation for the absence of impact is cultural norms and the values underlying these norms. In sub-Saharan and South Asian societies, children are valued, and women's status depends on their fertility. These factors could challenge transformation into capabilities because of the social consequences (gender-based violence, divorce, separation, stigmatization) if an individual woman challenges these societal norms. In our study, although women expressed the challenges of raising children, they continued to have children perhaps to avoid these potential social consequences of not doing so and to secure their social status (being a woman). We observed the same dynamic for the division of labor. Although women were aware of the inequality underlying the labor division, they accepted it against their will, probably to secure their marital status and vulnerable financial situation. These findings highlight the importance of considering the values underlying the concepts of women's status and cultural division of labor when examining the concept of women's capability [40].

The flexibility of the framework allowed us to see that introducing a new resource (women's group intervention) into women's capability space contributed to opening-up new opportunities for some, but not all women, and not in a uniform way. The cases of the woman who did not want to have more children but finally gave-in because of the pressure from her husband, and the woman who felt unsupported by her husband but remained with him, illustrate this. As pointed out by Kabeer, individual agency to challenge gender inequality cannot be addressed by individuals alone. There is a need for more collective political actions to tackle gender inequalities [40].

The Sen capability framework sheds light on what women aspire to and identifies a set of factors that can obstruct or facilitate the transformation of a resource into capabilities. Our study provides insights on the potential pathways that lead the shift from a resource into capabilities. Our findings suggest that knowledge and the approach to learning through discussion, reflection in groups, critical consciousness (realization of the ability to address mother and child's health issues), and support (social and financial) contributed to changing women's health capabilities. An emerging body of evidence describes similar mechanisms triggered by participatory women's group interventions, but more research is needed to clarify and unpack these pathways [6, 7, 11].

## Limitations

The project received external funding. The team members who collected the data are part of the project team, increasing the potential for social desirability bias. To minimize this bias, we explained to the participants that the study results would not impact the project and its funding. We paid attention to negative cases that seemed to contradict the emerging explanations to refine our analysis.

The limited number of interviews and focus group discussions may reduce generalizability to other Uganda districts and more widely. Studying gender dynamics requires exploring both gender's perspectives. A limitation of our study is that we did not interview men. It would be useful to confirm the extent of the perceived women's achievements using quantitative methods.

## Conclusion

This study aimed to explore the impact of a participatory community intervention on women's capability. Women could transform the resources from this intervention into more agency that led them to adopt safe and healthy behaviors for themselves during pregnancy and childbirth and for their children. However, women still faced poverty, poor access to economic opportunities, and gender inequalities, limiting their agency to expand freedoms to live the life they want to lead.

The intersection of access to economic opportunities, individual histories, gender norms, and the values underlying status concepts influenced women's capabilities. To expand women's freedoms, we need more political and collective actions to reduce gender inequalities and question the values underlying women's social status.

## Supplementary information

**Additional file 1.** Interview Guide.

**Additional file 2.** Focus Group guide.

## Data Availability

The datasets used and analyzed during the current study are available from the corresponding author on reasonable request.

## Abbreviations

FGD: Focus group discussion
ID: Individual interview
MocheLaSS: Mother–child health Lacor South Sudan
PLA: Participatory learning action
