# Supplementary material for: Exploring the impact of a community participatory intervention on women's capability: a qualitative study in Gulu Northern Uganda
Source: BMC Womens Health. 2021 Jan 18;21:28. doi: 10.1186/s12905-020-01170-8 (PMC7812725; doi:10.1186/s12905-020-01170-8)
Supplement: Supplementary file 1 — Additional file 1. Interview Guide. [file 12905_2020_1170_MOESM1_ESM.docx]

Additional file 1. Interview Guide

Name:

Code:

Age:

Occupation:

Level of education:

Religion:

Location (name of the village/area (Opit, Amuru, Lacor):

Name of the saving group:

Marital status:

In case of polygamy (number of co-wives):

Number of children:

**Being part of a women group**

- How did you decide to attend women's meeting groups for Mochelass project?
- What do you think about this project?
- How do you feel personally since you are a member of this group?
- What does your husband think about your participation in these meetings?

**Education**

- How many years of school have you achieved?
- What stops you from continuing your schooling?
- When you were at school, what was your job's dream?

**Since you are attending women's group's meeting, what changes have you seen in your life?**

**Division of labor**

- Can you tell me what you do since you wake up in the morning until you go to bed?
- What are your husband's tasks inside and outside the house?
- What do you think about each other tasks (husband and wife)?

**Resources**

- What resources can you claim that it is your own?
- What does your husband own?

**Health**

- When you do not feel well, what do you do?
- How do you manage your sickness?
- How do you manage your children sickness?

**Pregnancy**

- For your last pregnancy, how did you use the health services?
- What was the role of your husband during your pregnancy, labor, and postpartum period?
- How would you like to see your husband involved?

**Family planning and child spacing**

- How did you decide about the number of children you want to have?
- What do you know about family planning services?
- How do you manage birth spacing?

**Agency**

- What decisions do you make without asking somebody?
- How do you take decisions within the household?
- What kind of things/ activities that you can do entirely freely without asking the permission of anybody?
- Where can you go without asking anybody?

**Values**

- What does it mean to be a "good woman" in Acholi culture?
- What does it mean to be a "good man" in Acholi culture?
- How are women valued?
- How are men valued?

**Achievements**

- From your perception, what is a realization/ an achievement that you are proud of?
- As a woman, what are your main objectives you would like to achieve/ realize in your life?
