# Supplementary material for: Exploring the impact of a community participatory intervention on women's capability: a qualitative study in Gulu Northern Uganda
Source: BMC Womens Health. 2021 Jan 18;21:28. doi: 10.1186/s12905-020-01170-8 (PMC7812725; doi:10.1186/s12905-020-01170-8)
Supplement: Supplementary file 2 — Additional file 2. Focus Group guide. [file 12905_2020_1170_MOESM2_ESM.docx]

**Additional file 2. Focus group guide**

Location (name of the village/area (Opit, Amuru, Lacor):

Name of the saving group:

Nb of participants:

- What do you think about Mochelass project?
- What do you think about your role as a facilitator?
- As a group, what changes have you seen since you implemented this project?
- What makes people change their health behaviors?
